# Utilizing tumor deposit count as a stratification criterion in revising TNM staging system for patients with colorectal cancer: a nomogram review study

**DOI:** 10.3389/fonc.2025.1605030

**Published:** 2025-08-25

**Authors:** Lifei Zhang, Yunna Ma, Jiantao Dong, Jianhui Cai

**Affiliations:** ^1^ Department of Surgery, Hebei Medical University, Shijiazhuang, Hebei, China; ^2^ Department of Gastrointestinal Surgery, Hebei General Hospital, Shijiazhuang, Hebei, China; ^3^ Department of Cardiovascular Medicine, Hebei General Hospital, Shijiazhuang, Hebei, China; ^4^ Department of Oncology and Immunotherapy, Hebei General Hospital, Shijiazhuang, Hebei, China; ^5^ Department of Research and Development (R&D), Yetem Biotech Corp. Ltd., Shijiazhuang, Hebei, China

**Keywords:** colorectal cancer, tumor deposit, SEER, TNM, nomogram

## Abstract

**Background:**

Tumor deposit (TD) is an independent risk factor associated with recurrence or metastasis for patients with colorectal cancer (CRC). The scenario in which both TD and lymph node metastasis (LNM) are positive is not clearly illustrated by the current TNM staging system. Simply treating one TD as one or two LNMs by a weighting factor is inappropriate. The aim of this study was to evaluate the prognostic impact of TD counts and revise TNM staging by utilizing TD count as a stratification criterion in patients with CRC.

**Methods:**

All the cases diagnosed with CRC between 2010 and 2019 were extracted from the Surveillance, Epidemiology, and End Results (SEER) database. Patients who met all inclusion criteria were grouped as TD=0, TD=1, TD=2, or TD≥3 based on their TD counts. To compare overall survival (OS) between groups, survival curves were plotted using Kaplan–Meier methods with log-rank tests. Utilizing TD count as a stratification criterion, the NM classification was regrouped. Using the Cox proportional hazards model, univariate and multivariate analyses were performed to identify significant factors in the revised TNM staging system. A nomogram was then created. The C-index, calibration plots, and receiver operating characteristic (ROC) curve were used to verify the model’s accuracy.

**Results:**

A total of 60,145 patients who satisfied all inclusion requirements were ultimately included in the datasets for analysis. Among them, 6,092 (10.1%) had TDs, with only 1,384 (22.7%) staged as N1c. The 3-year OS rates were 74.6%, 50.4%, 39.8%, and 29.1% in the TD=0, TD=1, TD=2, and TD≥3 groups, respectively (p < 0.001), indicating that a greater TD count was linked to a worse prognosis. We introduced a novel stage, N2c, for patients with 10 or more LNMs. We found a striking overlap between the survival curves of this new subgroup and those of the M1a group, with p = 0.39. We observed that the survival trajectories of CRC patients with more than three TDs were similar to those of patients in the N2c group and the M1a group, with p = 0.15 and p = 0.42, respectively, which means that CRC patients with more than 10 LNMs or three TDs are equivalent to the presence of distant metastases. Utilizing TD count as a stratification criterion, we regrouped the NM classification. We finally assigned CRC patients with three or more TDs to the M1a. Finally, the revised TNM staging’s prognostic significance was demonstrated by the nomogram and dynamic nomogram. The C-indices for OS prediction in the training cohort and validation cohort were 0.751 (95% CI: 0.748–0.754) and 0.752 (95% CI: 0.747–0.756), respectively. The ROC curve study revealed that both the training cohort and the validation group had areas under the curve (AUCs) of approximately 0.8 at 1, 3, and 5 years. The calibration curves demonstrated good agreement between actual observation and the nomogram-predicted Cancer-Specific Death (CSD) probability. The clinical application study demonstrated that the model outperformed the TNM staging approach in terms of net benefit increases and fewer needless procedures.

**Conclusions:**

TDs have significant predictive significance in CRC. The revised TNM staging using TD counts as a stratification criterion predicts survival more accurately than the current staging. CRC patients with more than 10 LNMs or three TDs have a level of malignancy comparable to the existence of distant metastases. The dynamic nomogram could assist medical practitioners in diagnosing, providing prognoses, and optimizing treatment strategies more quickly and effectively.

## 1 Background

Optimizing cancer treatment strategies requires accurate prognostic assessment (1, 2). The most extensively used approach for colorectal cancer prognostic evaluation is the American Joint Committee on Cancer (AJCC) TNM staging system (3). It has changed dramatically over the last two decades, with the primary driver being the introduction of tumor deposit (4–6). The eighth edition of the AJCC staging system classified tumor deposit (TD) as a distinct tumor nodule that was found in the adjacent mesentery or pericolonic or perirectal fat and did not exhibit histological evidence of a remnant lymph node or a readily discernible vascular or neural component (7). N1c refers to colorectal cancer (CRC) individuals who have TD but no lymph node metastases (LNMs). In contrast, the presence of TD or LNM with any T lesion is defined as stage III, which means a local advanced stage that needs more aggressive treatment decisions (8). Nevertheless, when coexisting with positive nodes, TDs are discarded in nodal staging (e.g., pN1a + 5 TDs = pN1a). This contradicts the evidence that TDs with node involvement had a worse outcome than N1c alone (9). The N1c category fails to recognize the synergistic disadvantage of TD+LNM coexistence, resulting in a staging blind spot. In addition, each TD is handled similarly (1 TD = 10 TDs in staging impact), and all N1c patients receive similar adjuvant therapy (7). The failure to elevate therapy for widespread TDs demonstrates a key gap in precision oncology. TD is an independent risk factor associated with recurrence or metastasis (10, 11). The main controversy is that the TNM staging system, at present, has lost a substantial amount of information regarding TD and should be revised.

To date, numerous researchers have worked diligently to elucidate the role of TD (12–15). Compared to TD-negative N2 patients, N1c patients had superior 5-year overall survival (OS) and disease-free survival (DFS), but they had inferior 5-year DFS compared to TD-negative N1 patients, according to a meta-analysis (16). The influence on CRC prognosis of TD positivity may be between N1 and N2. Given that patients who tested positive for TD had a worse prognosis, several studies have demonstrated that TD should be distinguished from LNM. According to Shi M et al., a significant number of TDs would make the survival of CRC patients poorer (17). Dae Hee Pyo et al. recommended treating one TD as one positive lymph node while counting in order to increase the predictive accuracy of TNM staging (18). Meanwhile, Wang S. et al. concluded that the eighth TNM staging system’s “N1c” classification is inferior to weighing one TD as two LNMs (19). However, each TD may vary from others in terms of size, shape, or contour. Simply treating one TD as one or two LNMs by a weighting factor is inappropriate. Previous research results need to be ameliorated.

This study aimed to quantify the independent prognostic significance of TD counts relative to LNM status in CRC, develop a revised TNM staging system integrating TD counts as a stratification criterion, and rigorously validate its predictive accuracy for CRC patients, ultimately bridging pathological insights with precision prognostication.

## 2 Methods

### 2.1 Patient selection

**Figure 1:**
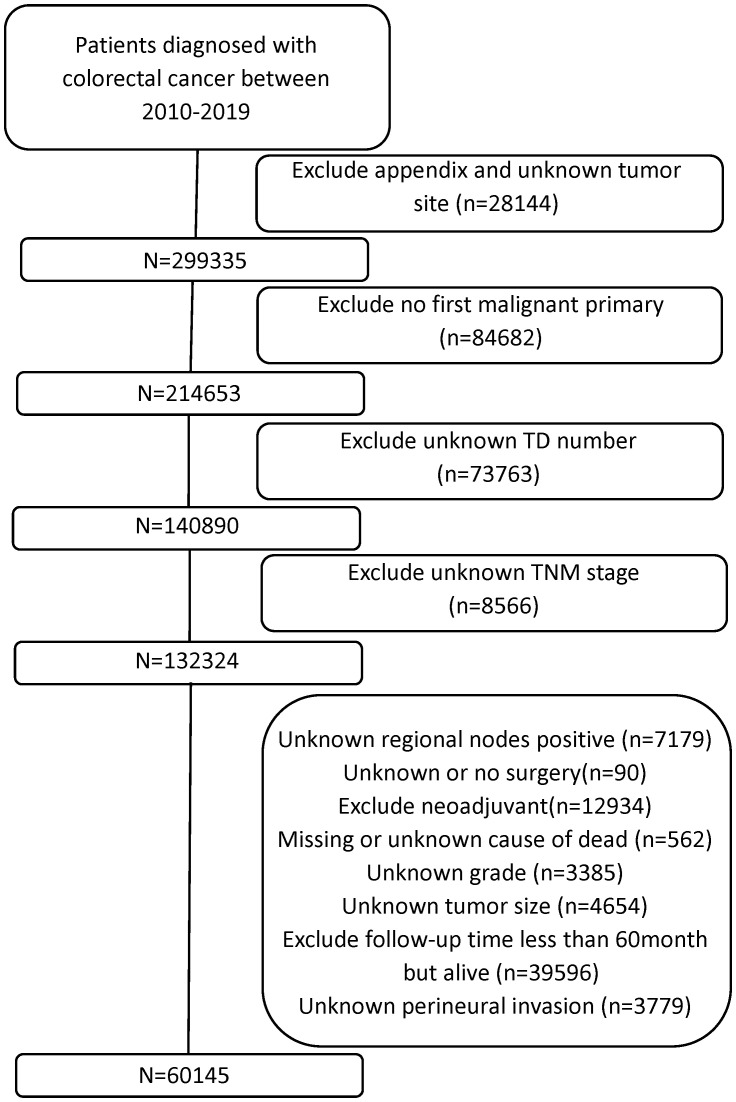
Flowchart of selection of study subjects.

Data of all patients with CRC diagnoses were collected from the SEER database [Research Data, 17 Registries, Nov 2023 Sub (2000–2021)] in April 2024 with reference number 22021-Nov2021. Using the SEER*Stat program version 8.4.4, we began with a nationwide cohort of 327,579 CRC cases from 2010 to 2019, identifying patients with the primary site code C18.0-C20.9. Some patients with the following exclusion criteria were excluded: 1) appendix and unknown tumor site, 2) no first malignant primary, 3) unknown TD number, 4) unknown TNM stage, 5) unknown regional nodes positive, 6) unknown or no surgery, 7) received neoadjuvant therapy, 8) missing or unknown cause of death, 9) unknown grade, 10) unknown tumor size, and 11) unknown perineural invasion. Patients with potential follow-up <60 months were excluded to ensure robust 5-year survival estimation. Cases recorded as “alive” with <60 months of follow-up represented data inconsistencies or eligibility violations and were removed. The research ultimately included 60,145 individuals who met all inclusion criteria. **Figure 1** shows the selection criteria and screening procedure.

### 2.2 Variables

The collected demographic and clinical variables included tumor deposit, age, gender, primary site, tumor size, grade, histology, AJCC T stage, AJCC N stage, AJCC M stage, radiation, chemotherapy, carcinoembryonic antigen (CEA) pretreatment, perineural invasion, marital status, survival time, and survival outcome.

Age was investigated as a continuous factor. Tumor deposit was divided into four groups: 0, 1, 2, and ≥3. Tumor sizes were split into groups of ≤50 and >50 mm. The two divisions were made using the best cut-off value determined by the Yale University-developed bioinformatics program X-Tile (version 3.6.1). Histology was classified into adenocarcinoma (ICD-O-3 code including 8140/3, 8210/3, 8211/3, 8213/3, 8221/3, 8260/3, 8261/3, 8262/3, and 8263/3), mucinous adenocarcinoma/signet ring cell carcinoma (ICD-O-3 code include 8480/3, 8481/3, and 8490/3), and others. OS was identified using the SEER “survival months” variable. The result of the follow-up was defined as either alive or dead from any cause.

### 2.3 Statistical analysis

To compare OS between groups, survival curves were created using log-rank testing and the Kaplan–Meier procedures. TNM staging was then revised by utilizing the TD count as a stratification criterion. The 7:3 ratio of eligible patients was used to randomly assign them to a validation cohort (n = 18,044) and a training group (n = 42,101). The baseline clinical variable features were compared using the Kruskal–Wallis H test for non-normally distributed continuous variables and the chi-square test for categorical data.

Using the Cox proportional hazards model, univariate and multivariate analyses were used to identify significant covariates (p < 0.05) in the training set. Variables’ calculated hazard ratios (HRs) were reported with 95% CI. Stepwise regression was used to determine the final model. Based on the findings of the multivariate analysis, the nomogram was constructed to predict the likelihood of individual survival. Then, a dynamic nomogram was created to use this model in clinical settings.

The nomogram’s capacity for discrimination was assessed using the bootstrap-corrected C-index. The ROC curves were created, together with the related areas under the curve (AUCs), building on the estimation that was previously presented. To do a more comprehensive assessment of the model’s calibration, the difference between the expected and actual probability was computed using the calibration plot.

For all data analyses, version 4.2.2 of the R program was utilized (http://www.R-project.org/). p-Values below 0.05 were regarded as statistically significant on both sides.

## 3 Results

### 3.1 Patient baseline characteristics

A total of 60,145 CRC patients were gathered from the SEER database, including 6,092 (10.1%) patients with TDs. Of the entire sets, 54,053 (89.9%) were classified as TD=0, 2,492 (4.1%) as TD=1, 1,283 (2.1%) as TD=2, and 2,317 (3.9%) as TD≥3. Among the 6,092 (10.1%) TD-positive CRC patients, only 1,384 (22.7%) were staged as N1c, comprising 797 as TD=1, 285 as TD=2, and 302 as TD≥3. All covariates, excluding gender, were significantly correlated with TD counts. The detailed information is shown in **Table 1**.

**Table 1:** Comparison of demographics and clinicopathologic characteristics based on the number of tumor deposits.

Variables	All patients (%)	TD=0 (%)	TD≥1 (%)	P	TD=1 (%)	TD=2 (%)	TD≥3 (%)	P
Total	60,145	54,053 (89.9)	6,092 (10.1)		2,492 (4.1)	1,283 (2.1)	2,317 (3.9)	
Age (median [IQR])	68.00 [57.00, 79.00]	68.00 [58.00, 79.00]	66.00 [56.00, 77.00]	<0.001	67.00 [56.00, 78.00]	67.00 [56.00, 77.00]	66.00 [55.00, 76.00]	<0.001
Gender								0.119
Female	30,256 (50.3)	27,253 (50.4)	3,003 (49.3)	0.099	1,262 (50.6)	619 (48.2)	1,122 (48.4)	
Male	29,889 (49.7)	26,800 (49.6)	3,089 (50.7)		1,230 (49.4)	664 (51.8)	1,195 (51.6)	
Primary site								<0.001
Right-side colon	32,557 (54.1)	29,499 (54.6)	3,058 (50.2)	<0.001	1,262 (50.6)	657 (51.2)	1,139 (49.2)	
Left-side colon	18,739 (31.2)	16,674 (30.8)	2,065 (33.9)		858 (34.4)	447 (34.8)	760 (32.8)	
Rectum	8,849 (14.7)	7,880 (14.6)	969 (15.9)		372 (14.9)	179 (14.0)	418 (18.0)	
Grade								<0.001
I	4,593 (7.6)	4,347 (8.0)	246 (4.0)	<0.001	98 (3.9)	56 (4.4)	92 (4.0)	
II	43,506 (72.3)	39,561 (73.2)	3,945 (64.8)		1,739 (69.8)	850 (66.3)	1,356 (58.5)	
III	9,835 (16.4)	8,352 (15.5)	1,483 (24.3)		529 (21.2)	298 (23.2)	656 (28.3)	
IV	2,211 (3.7)	1,793 (3.3)	418 (6.9)		126 (5.1)	79 (6.2)	213 (9.2)	
Histology								<0.001
Adenocarcinoma	54,038 (89.8)	48,732 (90.2)	5,306 (87.1)	<0.001	2,239 (89.8)	1,130 (88.1)	1,937 (83.6)	
Mucin/ring	5,206 (8.7)	4,592 (8.5)	614 (10.1)		202 (8.1)	118 (9.2)	294 (12.7)	
Other	901 (1.5)	729 (1.3)	172 (2.8)		51 (2.0)	35 (2.7)	86 (3.7)	
AJCC T stage								<0.001
T1	6,417 (10.7)	6,357 (11.8)	60 (1.0)	<0.001	39 (1.6)	12 (0.9)	9 (0.4)	
T2	9,202 (15.3)	8,984 (16.6)	218 (3.6)		128 (5.1)	44 (3.4)	46 (2.0)	
T3	33,059 (55.0)	29,744 (55.0)	3,315 (54.4)		1,507 (60.5)	698 (54.4)	1,110 (47.9)	
T4a	7,371 (12.3)	5,629 (10.4)	1,742 (28.6)		556 (22.3)	370 (28.8)	816 (35.2)	
T4b	4,096 (6.8)	3,339 (6.2)	757 (12.4)		262 (10.5)	159 (12.4)	336 (14.5)	
AJCC N stage								<0.001
N0	33,215 (55.2)	33,215 (61.4)	0 (0.0)	<0.001	0 (0.0)	0 (0.0)	0 (0.0)	
N1a	7,090 (11.8)	6,299 (11.7)	791 (13.0)		408 (16.4)	156 (12.2)	227 (9.8)	
N1b	7,813 (13.0)	6,528 (12.1)	1,285 (21.1)		512 (20.5)	285 (22.2)	488 (21.1)	
N1c	1,384 (2.3)	0 (0.0)	1,384 (22.7)		797 (32.0)	285 (22.2)	302 (13.0)	
N2a	5,359 (8.9)	4,175 (7.7)	1,184 (19.4)		412 (16.5)	271 (21.1)	501 (21.6)	
N2b	5,284 (8.8)	3,836 (7.1)	1,448 (23.8)		363 (14.6)	286 (22.3)	799 (34.5)	
AJCC M stage								<0.001
M0	51,925 (86.3)	48,189 (89.2)	3,736 (61.3)	<0.001	1,779 (71.4)	794 (61.9)	1,163 (50.2)	
M1a	5,095 (8.5)	3,846 (7.1)	1,249 (20.5)		434 (17.4)	267 (20.8)	548 (23.7)	
M1b	3,125 (5.2)	2,018 (3.7)	1,107 (18.2)		279 (11.2)	222 (17.3)	606 (26.2)	
Tumor size (mm)								<0.001
≤50	38,397 (63.8)	34,994 (64.7)	3,403 (55.9)	<0.001	1,420 (57.0)	711 (55.4)	1,272 (54.9)	
>50	21,748 (36.2)	19,059 (35.3)	2,689 (44.1)		1,072 (43.0)	572 (44.6)	1,045 (45.1)	
Radiation								<0.001
No	57,870 (96.2)	52,129 (96.4)	5,741 (94.2)	<0.001	2,359 (94.7)	1,215 (94.7)	2,167 (93.5)	
Yes	2,275 (3.8)	1,924 (3.6)	351 (5.8)		133 (5.3)	68 (5.3)	150 (6.5)	
Chemotherapy								<0.001
No	39,610 (65.9)	37,129 (68.7)	2,481 (40.7)	<0.001	1,074 (43.1)	524 (40.8)	883 (38.1)	
Yes	20,535 (34.1)	16,924 (31.3)	3,611 (59.3)		1,418 (56.9)	759 (59.2)	1,434 (61.9)	
CEA pretreatment								<0.001
Normal	20,352 (33.8)	18,916 (35.0)	1,436 (23.6)	<0.001	641 (25.7)	324 (25.3)	471 (20.3)	
Elevated	15,763 (26.2)	13,300 (24.6)	2,463 (40.4)		947 (38.0)	475 (37.0)	1,041 (44.9)	
Unknown	24,030 (40.0)	21,837 (40.4)	2,193 (36.0)		904 (36.3)	484 (37.7)	805 (34.7)	
Perineural invasion								<0.001
Negative	52,479 (87.3)	48,507 (89.7)	3,972 (65.2)	<0.001	1,843 (74.0)	869 (67.7)	1,260 (54.4)	
Positive	7,666 (12.7)	5,546 (10.3)	2,120 (34.8)		649 (26.0)	414 (32.3)	1,057 (45.6)	
Marital status								0.008
Married	31,031 (51.6)	28,015 (51.8)	3,016 (49.5)	0.001	1,229 (49.3)	641 (50.0)	1,146 (49.5)	
Unmarried	29,114 (48.4)	26,038 (48.2)	3,076 (50.5)		1,263 (50.7)	642 (50.0)	1,171 (50.5)	

AJCC, American Joint Committee on Cancer.

### 3.2 Survival analysis

**Figure 2:**
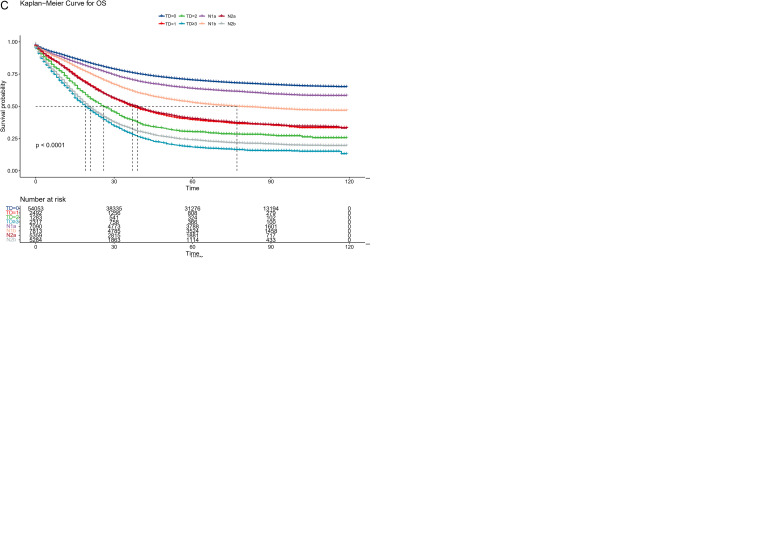
Comparison of overall survival among the current TNM groups. **(A)** Kaplan–Meier curves according to the tumor deposit counts. **(B)** Survival comparison between TD-positive and N1c CRC patients. **(C)** Survival comparison between TD groups and AJCC TNM stage groups. TD, tumor deposit; CRC, colorectal cancer; AJCC, American Joint Committee on Cancer.

For the eligible patients, the median follow-up period was 64 months (Interquartile Range (IQR): 20–87). A total of 31,416 patients (52.2%) died. The TD groups’ OS rates were contrasted (**Figure 2A**). TD count was a significant predictive factor for CRC patients, as seen by the 3-year OS rates of 74.6%, 50.4%, 39.8%, and 29.1% in the TD=0, TD=1, TD=2, and TD≥3 groups, respectively (p < 0.001). According to the AJCC TNM staging method, N1c stands for TD-positive patients. Thus, a survival analysis comparing the TD-positive group and the N1c group was conducted (**Figure 2B**). As indicated by the 3-year OS rates of 40.0% and 58.9% (p < 0.001), the N1c group was unable to prognostically predict the outcome for patients with TDs. Then, survival analysis was conducted between the TD groups and AJCC TNM stage groups (**Figure 2C**). The 3-year OS rates were 74.6%, 51.4%, 50.4%, 41.5%, 39.8%, 28.8%, 32.9%, and 29.1% in the TD=0, N2a, TD=1, T4b, TD=2, N2b, M1a, and TD≥3 groups, respectively (p < 0.001). The Kaplan–Meier curves of the TD=1 and N2a groups exhibited great similarity. The TD=2 group, however, was situated between the T4b and N2b groups. After the crossover, the TD≥3 and M1a groups partially merged.

### 3.3 Revised TNM staging

**Figure 3:**
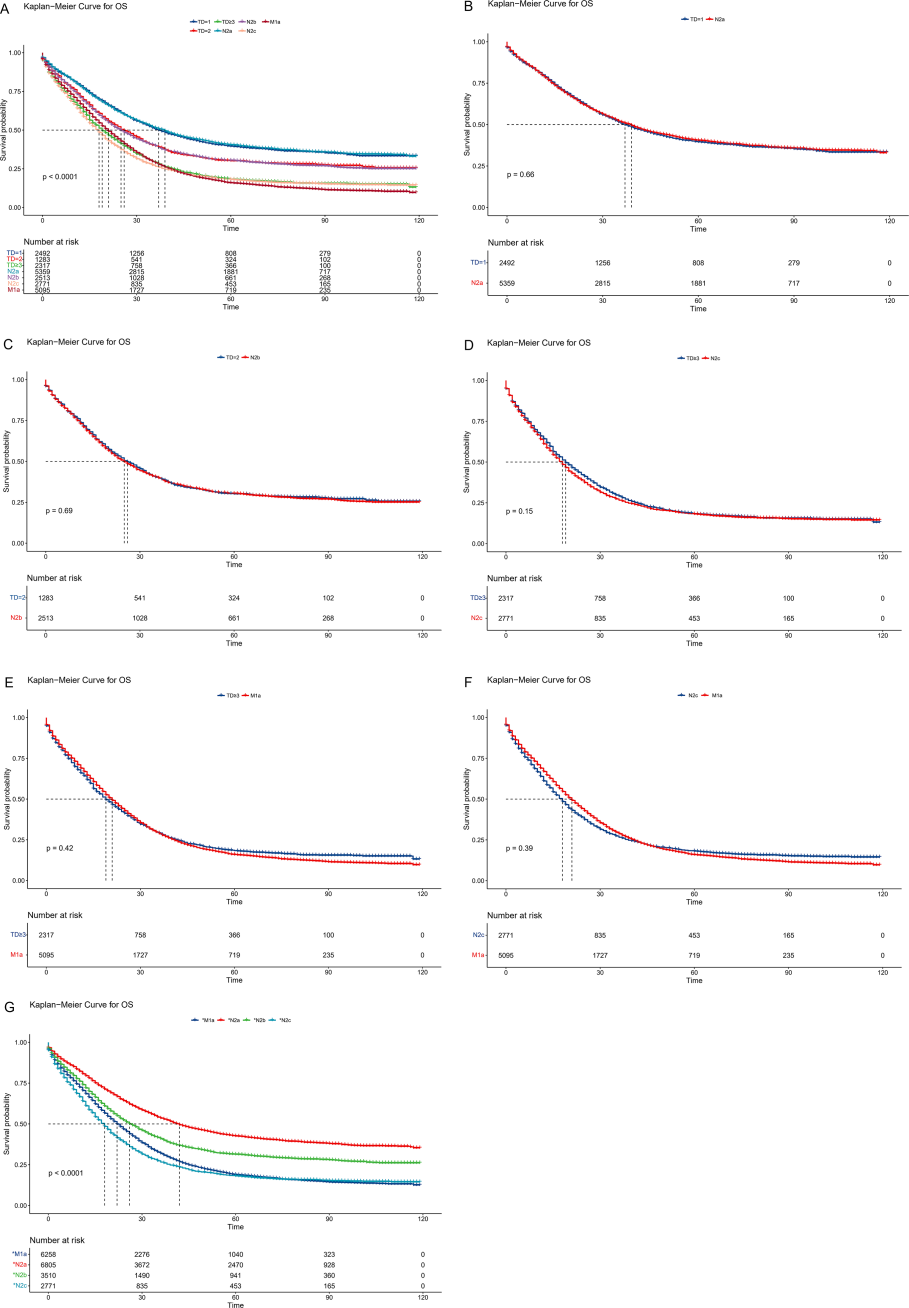
Comparison of overall survival among the revised TNM staging system. **(A)** Survival comparison between TD groups and regrouped new NM groups. **(B)** Survival comparison between TD=1 and new N2a couple. **(C)** Survival comparison between TD=2 and new N2b couple. **(D)** Survival comparison between TD≥3 and new N2c couple. **(E)** Survival comparison between TD≥3 and new M1a couple. **(F)** Survival comparison between M1a and new N2c couple. **(G)** Survival comparison of revised NM groups. TD, tumor deposit.

To establish a more scientific and reasonable TNM staging system, first, the TD count was considered a stratification criterion, and then the NM classification was regrouped. Unlike the current AJCC staging system, the N1c stage was eliminated, new N2a was regrouped as four to six LNMs, new N2b was regrouped as seven to nine LNMs, and the new N2c was created, which had 10 or more LNMs. Then, survival analysis was validated between the TD groups and the regrouped new NM groups (**Figure 3A**). The 3-year OS rates were 51.4%, 50.4%, 39.8%, 39.8%, 29.1%, 28.8%, and 26.6% in the new N2a, TD=1, new N2b, TD=2, TD≥3, M1a, and new N2c groups, respectively (p < 0.001). The Kaplan–Meier curves almost overlapped between the TD=1 and new N2a groups, similar to the TD=2 and new N2b groups. Meanwhile, the TD≥3 group was similar to the new N2c and M1a groups. Moreover, survival analysis was conducted between single couples (**Figures 3B–F**). The p-values were 0.66, 0.69, 0.42, 0.15, and 0.39 in the TD=1 new N2a couple, TD=2 new N2b couple, TD≥3 M1a couple, TD≥3 new N2c couple, and new N2c M1a couple, respectively. In the subgroup analysis, the TD≥3 and M1a couple exhibited greater similarity than the TD≥3 and new N2c couple, while the new N2c group had a similar malignancy to the M1a group. Finally, a revised TNM staging was constructed. TD=1 and new N2a were regrouped as *N2a, TD=2 and new N2b were regrouped as *N2b, TD≥3 and M1a were regrouped as *M1a, and new N2c was renamed as *N2c.

In the revised TNM staging system, 5,485 patients were adjusted from the N stage to *N2a(1717), *N2b(997), and *N2c(2771), whereas 1,163 patients were changed from the M0 stage to the *M1a stage (**Table 2**). The 3-year OS rates were 53.5%, 40.9%, 26.6%, and 31.9% in the *N2a, *N2b, *N2c, and *M1a stages, respectively (p < 0.001) (**Figure 3G**). For the current and updated TNM staging, the corresponding C-indices were 0.6731 (95% CI: 0.6715–0.6747) and 0.6744 (95% CI: 0.6728–0.6760).

**Table 2:** Stage migration in the revised staging system utilizing TD count as a stratification criterion.

/			AJCC 8th								
			N stage						M stage		
			N0 (LMNs = 0)	N1a (LMNs = 1)	N1b (LMNs = 2–3)	N1c (LMNs = 0/TD+)	N2a (LMNs = 4–6)	N2b (LMNs ≥ 7)	M0	M1a	M1b
Revised	N stage	N0 **(LMNs = 0)**	33,215	0	0	302	0	0	/	/	/
		N1a **(LMNs = 1)**	0	6,526	0	0	0	0	**/**	**/**	**/**
		N1b **(LMNs =2–3)**	0	0	7,016	0	0	0	/	/	/
		*N2a **(LMNs = 4–6/TD=1)**	0	**408**	**512**	**797**	5,088	0	**/**	**/**	**/**
		*N2b **(LMNs = 7–9/TD=2)**	0	**156**	**285**	**285**	**271**	2,513	/	/	/
		*N2c **(LMNs ≥ 10)**	0	0	0	0	0	**2,771**	**/**	**/**	**/**
	M stage	M0	31,966	5,912	5,901	875	3,451	2,657	50,762	0	0
		*M1a **(M1a/TD≥3)**	**/**	**/**	**/**	**/**	**/**	**/**	**1,163**	5,095	0
		M1b	/	/	/	/	/	/	0	0	3,125

AJCC, American Joint Committee on Cancer.

*Denotes the revised stages; bold font indicates the up-staged patients.

### 3.4 Univariate and multivariate analyses

**Table 3** shows the demographics and tumor features of the CRC patient group with the revised TNM staging. These data are presented in the entire set (n = 60,145), training set (n = 42,101), and validation set (n = 18,044).

**Table 3:** Baseline demographics and clinical characteristics for the cohort with revised TNM staging.

Variables	All patients (%)	Training cohort (%)	Validation cohort (%)	P
Total	60,145	42,101	18,044	
Age (median [IQR])	68.00 [57.00, 79.00]	68.00 [58.00, 79.00]	68.00 [57.00, 78.00]	0.548
Gender (%)				0.613
Female	30,256 (50.3)	21,150 (50.2)	9,106 (50.5)	
Male	29,889 (49.7)	20,951 (49.8)	8,938 (49.5)	
Primary site (%)				0.737
Right-side colon	32,557 (54.1)	22,788 (54.1)	9,769 (54.1)	
Left-side colon	18,739 (31.2)	13,146 (31.2)	5,593 (31.0)	
Rectum	8,849 (14.7)	6,167 (14.6)	2,682 (14.9)	
Grade (%)				0.647
I	4,593 (7.6)	3,209 (7.6)	1,384 (7.7)	
II	43,506 (72.3)	30,515 (72.5)	12,991 (72.0)	
III	9,835 (16.4)	6,841 (16.2)	2,994 (16.6)	
IV	2,211 (3.7)	1,536 (3.6)	675 (3.7)	
Histology (%)				0.103
Adenocarcinoma	54,038 (89.8)	37,801 (89.8)	16,237 (90.0)	
Mucin/ring	5,206 (8.7)	3,691 (8.8)	1,515 (8.4)	
Other	901 (1.5)	609 (1.4)	292 (1.6)	
AJCC T stage (%)				0.953
T1	6,417 (10.7)	4,489 (10.7)	1,928 (10.7)	
T2	9,202 (15.3)	6,466 (15.4)	2,736 (15.2)	
T3	33,059 (55.0)	23,142 (55.0)	9,917 (55.0)	
T4a	7,371 (12.3)	5,154 (12.2)	2,217 (12.3)	
T4b	4,096 (6.8)	2,850 (6.8)	1,246 (6.9)	
Revised N stage (%)				0.815
N0	33,517 (55.7)	23,400 (55.6)	10,117 (56.1)	
N1a	6,526 (10.9)	4,608 (10.9)	1,918 (10.6)	
N1b	7,016 (11.7)	4,917 (11.7)	2,099 (11.6)	
N2a	6,805 (11.3)	4,761 (11.3)	2,044 (11.3)	
N2b	3,510 (5.8)	2,458 (5.8)	1,052 (5.8)	
N2c	2,771 (4.6)	1,957 (4.6)	814 (4.5)	
Revised M stage (%)				0.469
M0	50,762 (84.4)	35,565 (84.5)	15,197 (84.2)	
M1a	6,258 (10.4)	4,379 (10.4)	1,879 (10.4)	
M1b	3,125 (5.2)	2,157 (5.1)	968 (5.4)	
Tumor size (mm)(%)				0.837
≤50	38,397 (63.8)	26,866 (63.8)	11,531 (63.9)	
>50	21,748 (36.2)	15,235 (36.2)	6,513 (36.1)	
Radiation (%)				0.191
No	57,870 (96.2)	40,480 (96.1)	17,390 (96.4)	
Yes	2,275 (3.8)	1,621 (3.9)	654 (3.6)	
Chemotherapy (%)				0.696
No	39,610 (65.9)	27,748 (65.9)	11,862 (65.7)	
Yes	20,535 (34.1)	14,353 (34.1)	6,182 (34.3)	
CEA pretreatment (%)				0.621
Normal	20,352 (33.8)	14,256 (33.9)	6,096 (33.8)	
Elevated	15,763 (26.2)	11,073 (26.3)	4,690 (26.0)	
Unknown	24,030 (40.0)	16,772 (39.8)	7,258 (40.2)	
Perineural invasion (%)				0.564
Negative	52,479 (87.3)	36,757 (87.3)	15,722 (87.1)	
Positive	7,666 (12.7)	5,344 (12.7)	2,322 (12.9)	
Marital status (%)				0.319
Married	31,031 (51.6)	21,665 (51.5)	9,366 (51.9)	
Unmarried	29,114 (48.4)	20,436 (48.5)	8,678 (48.1)	

AJCC, American Joint Committee on Cancer.

According to univariate analysis, age, primary site, tumor size, grade, histology, AJCC T stage, revised N stage, revised M stage, radiation, chemotherapy, CEA pretreatment, perineural invasion, and marital status were linked to OS (p < 0.05). The criteria for the final model were the stepwise regression’s least Akaike information criterion (AIC) score. The multivariate analysis and prediction model incorporated all additional covariates, with the exception of original site, histology, tumor size, and radiation. The findings of the univariate and multivariate Cox regression analyses for OS in patients with CRC are shown in **Table 4**, along with the HR and 95% CI.

**Table 4:** Univariate and multivariate analyses of CSD in the training set (n = 42,101).

Characteristic	Univariate analysis		Multivariate analysis	
	HR (95% CI)	P-value	HR (95% CI)	P-value
Age (median [IQR])	1.04 (1.04–1.04)	<0.001	1.04 (1.04–1.04)	<0.001
Gender (%)				
Female	Reference			
Male	1.02 (0.99–1.05)	0.133		
Primary site				
Right-side colon	Reference			
Left-side colon	0.83 (0.8–0.85)	<0.001		
Rectum	0.71 (0.68–0.74)	<0.001		
Grade				
I	Reference		Reference	
II	1.26 (1.19–1.33)	<0.001	1.04 (0.98–1.10)	0.172
III	1.95 (1.83–2.07)	<0.001	1.15 (1.08–1.22)	<0.001
IV	2.27 (2.09–2.46)	<0.001	1.29 (1.19–1.40)	<0.001
Histology				
Adenocarcinoma	Reference			
Mucin/ring	1.28 (1.22–1.34)	<0.001		
Other	2.03 (1.84–2.23)	<0.001		
AJCC T stage				
T1	Reference		Reference	
T2	1.4 (1.3–1.5)	<0.001	1.21 (1.13–1.30)	<0.001
T3	2.48 (2.34–2.64)	<0.001	1.73 (1.63–1.84)	<0.001
T4a	5.15 (4.83–5.5)	<0.001	2.68 (2.50–2.88)	<0.001
T4b	5.07 (4.73–5.44)	<0.001	2.83 (2.63–3.05)	<0.001
Revised N stage				
N0	Reference		Reference	
N1a	1.35 (1.29–1.41)	<0.001	1.38 (1.32–1.45)	<0.001
N1b	1.67 (1.6–1.74)	<0.001	1.62 (1.55–1.69)	<0.001
N2a	2.21 (2.12–2.3)	<0.001	1.90 (1.81–1.98)	<0.001
N2b	2.88 (2.74–3.02)	<0.001	2.25 (2.13–2.38)	<0.001
N2c	4.02 (3.82–4.23)	<0.001	2.74 (2.59–2.91)	<0.001
Revised M stage				
M0	Reference		Reference	
M1a	3.43 (3.31–3.55)	<0.001	2.73 (2.62–2.84)	<0.001
M1b	5.07 (4.84–5.32)	<0.001	3.54 (3.36–3.73)	<0.001
Tumor size (mm)				
≤50	Reference			
>50	1.51 (1.47–1.55)	<0.001		
Radiation				
No	Reference			
Yes	0.83 (0.77–0.89)	<0.001		
Chemotherapy				
No	Reference		Reference	
Yes	0.96 (0.94–0.99)	0.006	0.59 (0.57–0.61)	<0.001
CEA pretreatment				
Normal	Reference		Reference	
Elevated	2.24 (2.17–2.32)	<0.001	1.43 (1.38–1.49)	<0.001
Unknown	1.52 (1.47–1.57)	<0.001	1.30 (1.26–1.35)	<0.001
Perineural invasion				
Negative	Reference		Reference	
Positive	1.99 (1.92–2.06)	<0.001	1.20 (1.16–1.24)	<0.001
Marital status				
Married	Reference		Reference	
Unmarried	1.52 (1.48–1.56)	<0.001	1.24 (1.21–1.28)	<0.001

HR, hazard ratio.

### 3.5 Prognostic nomogram construction and validation

**Figure 4:**
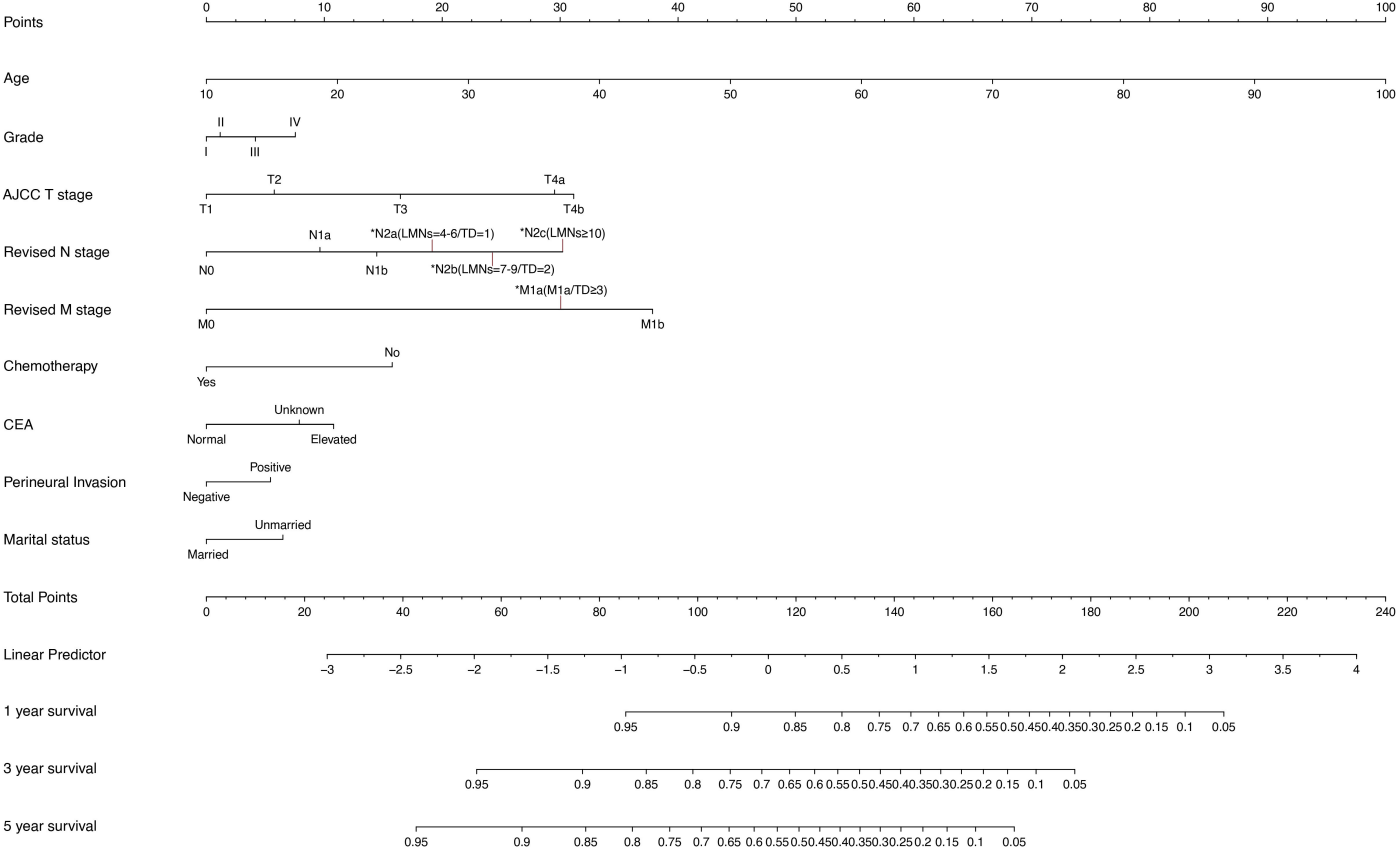
Nomogram for predicting 1-, 3-, and 5-year OS. OS, overall survival.

To predict OS at 1, 3, and 5 years in CRC patients, a nomogram was developed using the multivariate model from the training cohort (**Figure 4**). The total score given at the bottom of the graph can be used to forecast the 1-, 3-, and 5-year OS rates of a particular patient by summing the scores for the individual elements in the nomogram, which summarizes the scores achieved on the scale for each of these risk variables.

**Figure 5:**
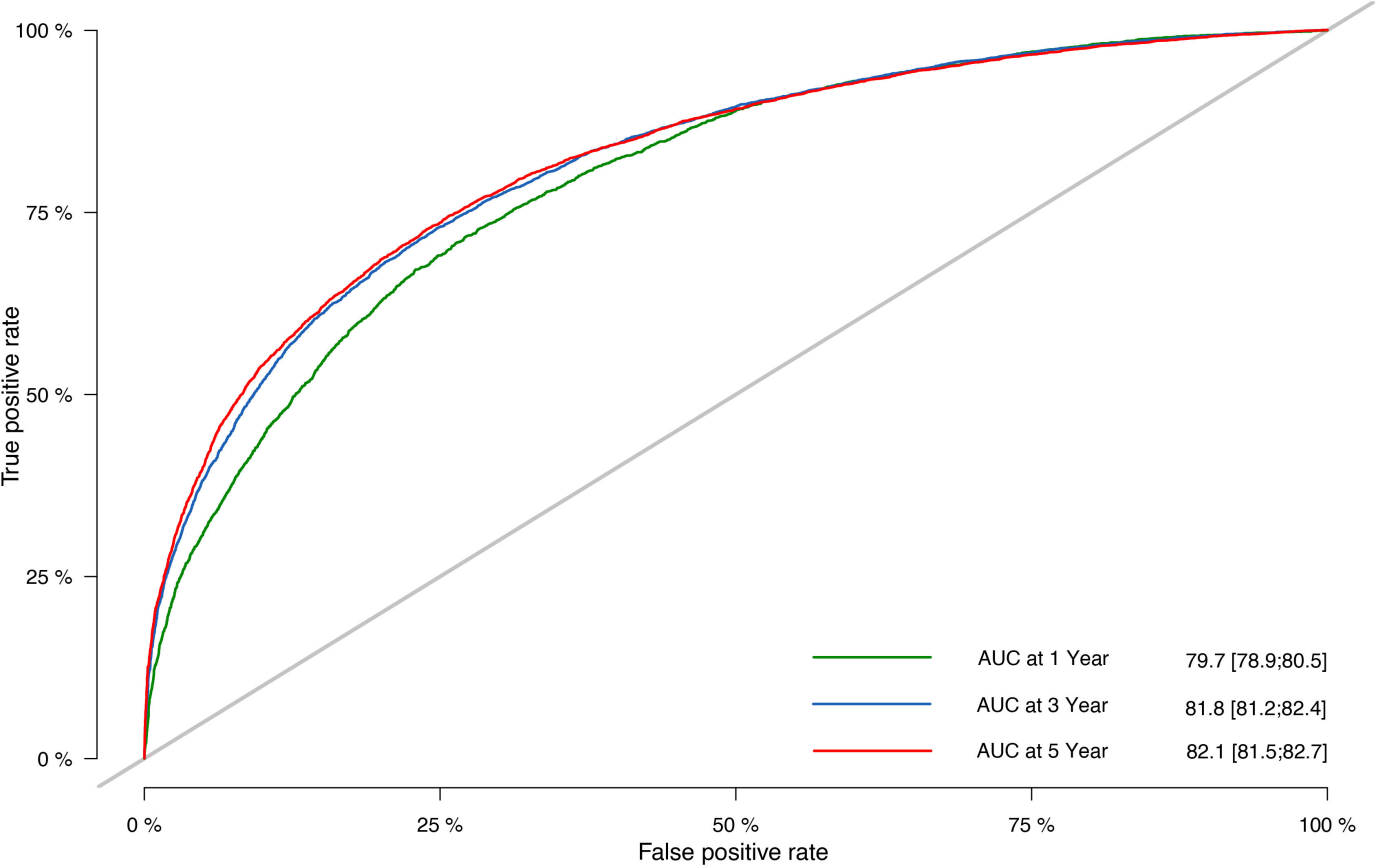
Nomogram ROC curves to predict 1-, 3-, and 5-year OS in the validation cohort. ROC, receiver operating characteristic; OS, overall survival.

The prognostic nomogram’s C-indices for OS prediction in the training and validation cohorts were 0.751 (95% CI: 0.748–0.754) and 0.752 (95% CI: 0.747–0.756), respectively. The ROC curves (**Figure 5**) were created in the same way as the C-index, and the corresponding AUC values were obtained. The AUC values for predicting 1-, 3-, and 5-year OS rates in the training cohort were 0.797 (95% CI: 0.792–0.803), 0.816 (95% CI: 0.812–0.820), and 0.823 (95% CI: 0.819–0.827), respectively. The validation cohort’s numbers, however, were 0.797 (95% CI: 0.789–0.805), 0.818 (95% CI: 0.812–0.824), and 0.821 (95% CI: 0.815–0.827). The results proved the predictive model’s remarkable accuracy.

**Figure 6:**
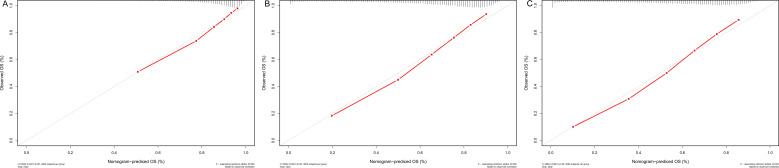
**(A–C)** Nomogram calibration plots to predict 1-,3-, and 5-year CSD in the validation cohort.

Meanwhile, **Figure 6** depicts the calibration curves for the probability of OS at 1, 3, and 5 years. The calibration plots were all rather close to the 45-degree diagonal line, showing the extraordinary accuracy of the predictions made using the nomogram models.

### 3.6 Dynamic nomogram construction

To make it easier and more practical for medical professionals to apply the developed nomogram in clinical settings, initially, we connected the RStudio application to a shinyapps server account that we had built. After that, four files were created using the multivariable Cox regression results: functions.R, global.R, server.R, and ui.R. The nomogram was made available online once all files had been submitted to the shinyapps server (https://tdrevisetnm.shinyapps.io/dynnomapp/). The clinical variables for the patient are listed on the website’s left side and can be entered by selecting an option next to each variable. After that, the survival rate can be calculated.

## 4 Discussion

The results of this study showed a high correlation between tumor aggressiveness characteristics and TD numbers, where a greater TD count was linked to a worse prognosis. Because of the lack of a description of the number of TDs, the N1c staging in the current eighth edition of the AJCC TNM staging method severely underestimates the influence of TDs on outcome. The revised TNM staging incorporates the number of afflicted regional lymph nodes, the degree of infiltration, and distant metastases, as well as a complete assessment of the impact of TD counts on recovery. Utilizing TD counts as a stratification criterion, 6,648 (11.1%) patients were up-staged, and the increased C-index indicates that the revised TNM staging produced a better accurate prognosis prediction than the present TNM staging.

Initially discovered in 1935, scientists have been studying TDs for ages. Evidence suggests two non-mutually exclusive origins of TD. 1) Vascular/perineural invasion: TD may arise from extramural vascular invasion (EMVI), where tumor clusters dislodge from primary sites and colonize perivascular/perineural spaces, lacking true lymphoid tissue (20, 21). 2) Obliterated lymph nodes: A subset likely represents replaced lymph nodes where tumor growth destroys nodal architecture (22–24). Even though our understanding of TD has improved in recent years, the mechanism of its presence and development is currently unclear. Studies have shown that TD, the early stage of distant metastasis, similar to intrahepatic metastasis of liver cancer and intrapulmonary metastasis of lung cancer, is caused by intra-organic spread of the original tumor (25). Chen J et al. claimed that the prevalence of TD in CRC patients can be independently predicted by primary site, T stage, grade, CEA, and LNMs (26), which is similar to the data in **Table 1** of this investigation. Overall, TD is more often associated with aggressive characteristics of the disease.

Additional research has been conducted to determine the prognostic significance of TD and explore its integration into the TNM staging system. In terms of prediction, the updated nodal staging approach by Li et al., which incorporates TDs as positive lymph nodes, performed better than the seventh edition of the AJCC staging system (27). Dae et al. hypothesized that the updated nodal staging, which combined TD counts with lymph nodes and introduced a novel N3 stage, may predict survival more precisely than current staging (18). However, other studies have shown that TD and LNM reporting should be different. In terms of biology and results, TDs are not equivalent to LNMs, per a meta-analysis (20). Wang S et al. preferred to weight one TD as two LNMs (19). We believe that TD should be characterized according to the level of malignancy. Therefore, based on the survival curves of groups with varying numbers of positive lymph nodes, we eliminated N1c staging and introduced N2c staging, which contained 10 or more positive lymph nodes, reassigning TD-positive colorectal cancer patients in accordance with the comparability of survival curves across groups with varied numbers of TDs and different numbers of LNMs.

In line with many previous experiments, we also found that the prognosis worsened with the number of LNMs (28, 29). N2c, a new stage, was created for patients with 10 or more LNMs. We found a striking overlap between the survival curves of this new subgroup and those of the M1a group, with p = 0.39, indicating no statistically significant difference. Meanwhile, we observed that the survival trajectories of CRC patients with more than three TDs were similar to those of patients in the N2c group and the M1a group, with p = 0.15 and p = 0.42, respectively, which means that in CRC patients, having more than 10 LNMs or three TDs is equivalent to the presence of distant metastases. This serves as a reminder that more intensive treatment and follow-up schedules are necessary for the two particular groups. Finally, groups with three or more TDs were assigned to the M1a rather than the N2c since the former had a slightly higher C-index when combined.

We propose TD≥3 reclassification as a biologically plausible, clinically actionable hypothesis and not a definitive conclusion. This approach aligns with TNM’s historical evolution, where prognostic data frequently precede comprehensive biological validation. For instance, the eighth edition of AJCC initially redefined T1a/T1b melanoma subtypes based solely on survival cutoffs (30). Similarly, the Union for International Cancer Control (UICC) gastric cancer staging classifies cytology-positive peritoneal washings as M1 without size criteria (31). Within this conceptual framework, grouping TD≥3 with M1a serves as a critical clinical alert for therapy intensification. Specifically, under current practice, TD≥3 patients receive stage III therapy, achieving only 29.1% 3-year OS. By contrast, metastasis-level regimens (e.g., those for oligometastatic disease) may improve OS, as extrapolated from clinical trials. Such redefinitions—grounded in alignment between survival outcomes and biological profiles—enable rational therapy escalation prior to randomized controlled trial (RCT) confirmation. Consequently, survival-driven staging revisions frequently accelerate clinical translation by preceding mechanistic studies.

Finally, the revised TNM staging’s prognostic significance was demonstrated by the nomogram and dynamic nomogram. The weighted scores in the nomogram revealed that the T, N, and M stages were the most closely related to patient prognosis in the revised TNM staging method, which was consistent with the prior traditional TNM staging approach (32, 33). Meanwhile, we discovered that the M1a group, more than the three TD groups and more than the 10 LNM groups, had the most similar weighted scores in the nomogram, supporting the three groups’ similar prognosis as previously stated. Chemotherapy was the fourth factor that influenced the outcome, highlighting the importance of designing uniform chemotherapy methods after surgery. The eighth edition of AJCC categorizes patients with TD≥3 as stage III (N1c if lymph nodes are negative), and the recommended treatment is adjuvant chemotherapy alone (e.g., CAPOX for 3 to 6 months) (7). However, our results demonstrate that these patients had a 3-year overall survival rate of 29.1%, which is comparable to that of M1a single metastatic illness (30.2%, p = 0.42), indicating that the current therapeutic method is inadequate. Reclassifying TD≥3 as M1a is reasonable from biological and prognosis perspectives. These individuals should be treated with combined chemotherapy and targeted medicines (e.g., bevacizumab), as is done for oligometastatic illness. Although retrospective results support this method, it is critical to validate it in prospective trials such as PRODIGE 42 before it is used in guidelines. In addition, marital status, which has a weighted score that is even higher than that of perineural invasion, should be included as another predictive parameter, reminding us that unmarried/single individuals should be given more attention. While marital status lacks direct biological causality with TD formation, it serves as a proxy for healthcare access and psychosocial stress. The higher TD risk in unmarried patients (**Table 1**) likely reflects the cumulative effects of diagnostic delays and stress-promoted tumor aggression. This association should not imply marital status as a therapeutic target. Rather, it highlights the need for social support interventions in high-risk groups. The variables determining prognosis in this study’s nomogram are broadly compatible with the results of other retrospective investigations, with just a minor variance in the weight of the influence of each factor (34, 35).

Our study demonstrates that TD count is a powerful, independent prognostic modifier in CRC. We recommend that 1) central pathology review and standardized TD reporting (including precise enumeration, anatomic location, and extranodal extension status) should be enforced in prospective trials to reduce inter-observer variability. 2) The dynamic nomogram should be externally validated in Asian and European datasets and then integrated into Electronic Health Record (EHR)-based decision support tools to facilitate personalized adjuvant therapy intensity and surveillance intervals. 3) Future research should explore the molecular landscape (e.g., consensus molecular subtype, tumor budding, and immune microenvironment) that underlies aggressive TD biology and should test whether TD-directed neoadjuvant therapy or anti-angiogenic agents can improve outcomes in this newly defined M1a-equivalent population.

## 5 Limitation

A number of limitations must also be considered. First, despite the huge sample size, given that this is a retrospective study, the patient selection strategy may be biased. Furthermore, using information from a public transparency database from the United States, we tested the nomogram in the preliminary study by allocating eligible cases randomly to the training and validation cohorts. More validation in an independent prospective cohort based on a distinct demographic is still needed before the study is expanded. In addition, the SEER database just records the number of TDs; however, there are no statistics on the parameters that may raise the weight of prognosis, such as size, location, and shape, which should be investigated further in future studies. Moreover, because the SEER database lacks extensive information about detailed chemotherapy regimen (e.g., FOLFOX vs. CAPOX, dosing intensity, or treatment duration) or molecular markers or some important clinical signs like smoking, evaluating their effects is difficult. These elements can potentially affect how well the nomograms work. In light of this, it may be possible to improve the nomogram’s prognostication capabilities in further studies by taking these parameters into account.

## 6 Conclusion

In conclusion, TDs have significant predictive significance in CRC and should not be simply equated with LNMs. The revised TNM staging using TD counts as a stratification criterion predicts survival more accurately than the current staging. CRC patients with more than 10 LNMs or three TDs have a level of malignancy comparable to the existence of distant metastases. The dynamic nomogram could assist medical practitioners in diagnosing, providing prognoses, and optimizing treatment strategies more quickly and effectively.

## Data Availability

The original contributions presented in the study are included in the article/supplementary material. Further inquiries can be directed to the corresponding author.
